# Decreasing initial telomere length in humans intergenerationally understates age-associated telomere shortening

**DOI:** 10.1111/acel.12347

**Published:** 2015-05-07

**Authors:** Brody Holohan, Tim De Meyer, Kimberly Batten, Massimo Mangino, Steven C Hunt, Sofie Bekaert, Marc L De Buyzere, Ernst R Rietzschel, Tim D Spector, Woodring E Wright, Jerry W Shay

**Affiliations:** 1 Department of Cell Biology, UT Southwestern Medical CenterDallas, TX, 75390, USA; 2 Department of Mathematical Modeling, Statistics and Bioinformatics, University of GhentGhent, 9000, Belgium; 3 Department of Twin Research and Genetic Epidemiology, King’s College LondonKing’s College London St. Thomas’ Hospital Campus South Wing, Block D, 3rd Floor Westminster Bridge Road, London, SE1 7EH, London, UK; 4 NIHR Biomedical Research Centre at Guy’s and St. Thomas’ Foundation TrustKing’s College London St. Thomas’ Hospital Campus South Wing, Block D, 3rd Floor Westminster Bridge Road, London, SE1 7EH, London, UK; 5 Cardiovascular Genetics Division, Department of Internal Medicine, University of UtahSalt Lake City, UT, 84108, USA; 6 Bimetra, Clinical Research Center Ghent, Ghent University HospitalGhent, Belgium; 7 Department of Cardiovascular Diseases, Ghent University Hospital, Ghent UniversityGhent, Belgium; 8 Center for Excellence in Genomics Medicine Research, King Abdulaziz UniversityJeddah, Saudi Arabia

**Keywords:** aging, genetics, human, parental effects, secular trend, telomeres, telomerase, telomere length

## Abstract

Telomere length shortens with aging, and short telomeres have been linked to a wide variety of pathologies. Previous studies suggested a discrepancy in age-associated telomere shortening rate estimated by cross-sectional studies versus the rate measured in longitudinal studies, indicating a potential bias in cross-sectional estimates. Intergenerational changes in initial telomere length, such as that predicted by the previously described effect of a father’s age at birth of his offspring (FAB), could explain the discrepancy in shortening rate measurements. We evaluated whether changes occur in initial telomere length over multiple generations in three large datasets and identified paternal birth year (PBY) as a variable that reconciles the difference between longitudinal and cross-sectional measurements. We also clarify the association between FAB and offspring telomere length, demonstrating that this effect is substantially larger than reported in the past. These results indicate the presence of a downward secular trend in telomere length at birth over generational time with potential public health implications.

## Introduction

The termini of linear chromosomes are shielded from recognition as DNA double-strand breaks by telomeres, TTAGGG repeats that recruit a group of protective protein accessory factors, the shelterin complex (de Lange, 2010). In humans, most normal somatic cells do not express telomerase, the enzyme capable of maintaining telomeres, a ribonucleoprotein reverse transcriptase. Therefore, normal telomerase-negative cells demonstrate progressive telomere shortening over time in all human somatic tissues (Wright *et al*., 1996). This eventually triggers cell growth arrest, termed ‘replicative senescence,’ via an uncapped telomere end recognized by the p53 pathway (Chin *et al*., 1999). Telomere shortening occurs both *in vitro* and *in vivo*, even in cell populations derived from telomerase-positive stem cell compartments such as leukocytes (Daniali *et al*., 2013). Leukocyte telomere length (LTL) shortens with age in humans, and reduced LTL has been linked to a host of pathologies such as dementia, hypertension, cardiovascular events, asthma, diabetes, and stroke as well as environmental factors, such as smoking, obesity, chronic infections, and stress (reviewed in Bojesen (2013)).

**Fig 1:**
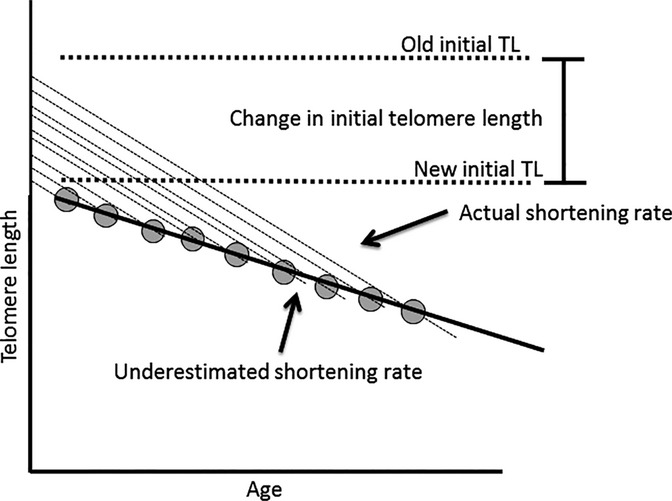
Decreasing initial telomere length understates telomere shortening rate. Cross-sectional analyses rely on the assumption that initial telomere length is not changing. In a cross-sectional estimate of telomere shortening rate (black line), the measurement of telomere length in multiple individuals of different ages (gray circles) will yield an underestimate of the actual telomere shortening rate (constant for all samples, dotted lines) if initial telomere length (y-intercept) is decreasing with time.

Because telomere length correlates with both these pathologies and environmental exposures known to drive them, telomere length may be an important indicator of general health, as an integrator of total physiological stress. Indeed, LTL may be a better indicator of biological age than chronological age and telomere measurements in leukocytes reflect systemic telomere length in other tissues (Daniali *et al*., 2013). Estimates of average telomere lengths for individuals of a given age as well as telomere shortening rates are predominantly based on cross-sectional analysis, large-scale measurements of individuals of differing ages. This estimation relies on the assumption that the average initial telomere length for all individuals measured does not change over time (e.g., from generation to generation). In cross-sectional studies, each individual is only measured once, so all differences in telomere length with age are ascribed to aging itself, even if generational changes have occurred. If a time-dependent change in initial telomere length, a secular trend, exists, the measurement of telomere shortening rate will be biased in the direction opposite the secular trend, leading to incorrect cross-sectional measurements of the rate of telomere shortening with age (Fig.1/Video S1). If initial/birth telomere length were increasing, cross-sectional analyses would indicate that telomere length shortens faster over time than it does in reality because younger individuals started with longer telomeres compared to older individuals, thus exaggerating age-associated differences. Conversely, if initial/birth telomere length were decreasing, cross-sectional analysis would generate aberrantly low estimates of attrition rate, as the difference between younger and older individuals would be understated. This important consideration about a secular trend in initial telomere length has not been adequately addressed.

A small number of studies have measured telomere attrition rate longitudinally by measuring LTL in a sample population and then measuring the same individuals again at a second later time point (Gardner *et al*., 2005; Aviv *et al*., 2009; Ehrlenbach *et al*., 2009; Farzaneh-Far *et al*., 2010; Chen *et al*., 2011; Houben *et al*., 2011). Shortening is then computed as the average difference between the first and second measurement of each individual in the population. Longitudinal estimates of telomere shortening consistently produce telomere attrition rates greater than cross-sectional shortening rates by ∼20 base pairs (bp) per year. Indeed, one longitudinal study compared cross-sectional analysis of their data at each telomere measurement time point and noted this discrepancy compared with their longitudinal analysis (Chen *et al*., 2011). Because longitudinal studies directly observe telomere shortening in individuals, one could argue that they represent a more correct measurement of the actual telomere shortening rate. These observations lead to the conclusion that something is biasing cross-sectional studies’ measurements. If a secular trend in initial telomere length were responsible for the bias, initial telomere length would be decreasing.

Population average telomere length demonstrably can change over evolutionarily short timescales, with differing mean LTL reported between ethnic groups (Aviv *et al*., 2009) and countries (Eisenberg *et al*., 2011). Given the substantial interindividual variation in LTL of several kilobases, telomere length could easily change as a result of founder effects, as well as a passenger trait during the fixation of beneficial variants. Telomere length is inherited both genetically, as a result of polymorphisms that confer changes in telomere length such as a number of SNPs identified in the TERT gene and epigenetica-lly, as the telomere repeats themselves are directly passed to the genome of the offspring (De Meyer *et al*., 2014). The dual inheritance mode of telomere length is most readily observed in telomeropathies, disorders of impaired telomere maintenance (Holohan *et al*., 2014). Offspring of individuals with subclinical or milder forms of these diseases inherit short telomeres directly from their parents as well as the genetic polymorphism responsible for the shortening, compounding the change in telomere length and decreasing the age of onset as well as increasing the severity of the diseases (Armanios *et al*., 2005). This dual inheritance may generate feed-forward loops in changes in telomere length, constrained by selection at the lower end by telomeropathies and incompletely understood fitness penalties for aberrantly long telomeres, such as an increased risk of a number of cancers (Anic *et al*., 2013).

Many important physiological and developmental parameters are the subject of secular (time-dependent) trends in the developed world, such as decreasing age at pubertal onset (Toppari & Juul, 2010), increasing rates of obesity/diabetes and hypertension, decreased rates of smoking (Romero *et al*., 2012), and increasing height (Komlos & Breitfelder, 2008). All of these trends are likely to impact telomere dynamics, as they correlate with the amount and timing of cell division, sex hormones (estrogen and testosterone), nutritional state, and growth signaling, as well as exposure to cigarette smoke, all of which are known to impact telomere biology. Given the nature of these trends, an alteration in initial telomere length or population-scale telomere dynamics is quite possible.

A number of groups have reported a positive association between a father’s age at the birth of their offspring (FAB) and LTL (Unryn *et al*., 2005; De Meyer *et al*., 2007; Aston *et al*., 2012; Eisenberg *et al*., 2012). This has been linked to a positive correlation between male age and sperm telomere length in a cross-sectional analysis, showing telomere elongation in sperm cell progenitors of roughly 57 bp/year (Aston *et al*., 2012). The average FAB in the developed world has been increasing in the recent past (Bray *et al*., 2006), which should produce an increase in initial LTL. If such an increase were occurring, cross-sectional analyses would report shortening rates higher than longitudinal studies, the opposite of the trend observed. If sperm progenitor cells instead maintained their telomere length at a fixed equilibrium length and that equilibrium length had been decreasing with time in younger men, this would be observed as an age-dependent increase in a cross-sectional measurement of sperm telomere dynamics. A decreasing equilibrium sperm telomere length over time would reconcile the discrepancy between demographic trends, sperm telomere dynamics, and the difference between longitudinal and cross-sectional LTL shortening rate.

Because in most studies, subject recruitment is mostly limited to a few years, an individual’s age and birth year are typically nearly perfectly collinear. Therefore, we decided to test in three large population datasets for a change in initial telomere length by determining whether an individual’s paternal birth year (PBY), an index for time that is less collinear with age, better predicts telomere length compared to FAB when age is included in the model. We then used mediation analysis to determine to what extent the measured effects of the three variables are influenced by their collinearity in these datasets. Lastly, we performed a ‘pseudo-longitudinal’ analysis of telomere shortening in one of the datasets that included twins. We performed these analyses in the UK twin adult registry (UK Twins), the National Heart Lung and Blood Institute Family Heart Study (NHLBI-FHS), and the Asklepios study populations, which included information on FAB, PBY, age, and telomere length in 2710, 2177, and 2434 individuals, respectively. PBY predicts telomere length better than FAB in all three datasets, and models that utilize PBY instead of FAB produce age-associated shortening rates more consistent with longitudinal telomere shortening rates, indicating that a negative secular trend in initial telomere length over time could partially explain the discrepancy. Furthermore, accounting for the collinearity between those variables reveals a heterogeneous PBY effect and a larger FAB effect than previously reported.

## Results

### Cross-sectional estimates of telomere shortening rate are consistently lower than longitudinal estimates

Table S1 (Supporting information) summarizes the most important longitudinal studies that measured telomere shortening rate in nonarbitrary units contrasted with studies that examined attrition rate cross-sectionally in large cohorts. The weighted average telomere shortening rate in cross-sectional studies is 22.82 base pairs per year compared with 41.19 in longitudinal studies (*P* < 0.001).

### Study characteristics for paternal birth year analyses

Telomere measurements and inclusion criteria for all three datasets have been described elsewhere (Higgins *et al*., 1996; Andrew *et al*., 2001; Rietzschel *et al*., 2007; Kimura *et al*., 2008). Paternal birth year (PBY) was collinear with age and father’s age at birth of the offspring, and age was collinear with FAB in all three datasets (Table S2), as were maternal variables; because of the collinearity between maternal and paternal effects, we could not distinguish if maternal variables contribute to the phenomena examined. Maternal effects were similar to paternal effects but of reduced statistical significance in the three populations; therefore, subsequent analyses were conducted only on paternal variables.

### Both paternal birth year and father’s age at birth influence telomere length

Linear regression with terminal restriction fragment (TRF, a gel-based measure of telomere length) length as the dependent variable and either FAB or PBY in addition to age as predictors was conducted on all three datasets individually and also on all three datasets combined. For the combined dataset analysis, a categorical variable indicating dataset was included to avoid bias from interpopulation differences in mean TRF length. Because of the size of the datasets, *P*-values for all effects reported as significant are <0.001 unless otherwise noted.

In all three datasets, models that included PBY instead of FAB better predicted telomere length, as demonstrated by their higher overall *R*^2^ values. PBY’s fractional *R*^2^ value was also higher than FAB’s, showing that it contributed more to the model’s ability to predict telomere length (Table1). Interestingly, age’s fractional *R*^2^ value substantially decreases in models that include PBY (0.0581 in the combined PBY model compared with 0.1629 in FAB), suggesting that the effect of age in other models is partially mediated by age’s highly collinear relationship with PBY. Furthermore, age-associated shortening rates become consistent with longitudinal attrition rates when PBY is included instead of FAB (39.44 bp/year in the PBY model compared with 22.18 bp/year in the FAB model). Omission of PBY from the model could explain why previous cross-sectional analyses have arrived at erroneously low estimates of age-associated telomere shortening.

**Table 1:** Comparison of paternal effect models shows that PBY can influence telomere length distinctly from FAB

Model	Study	Paternal effect (95% CI) (bp/year)	Age effect (95% CI) (bp/year)	Paternal effect partial *R*^2^	Age effect partial *R*^2^	Model *R*^2^
Paternal birth year (PBY)	UK Twins	−19.48 (−23.03 to −15.93)	−40.94 (−45.04 to −36.82)	0.0681	0.1144	0.1825
	NHLBI-FHS	−14.66 (−18.18 to −11.14)	−36.42 (−40.54 to −32.31)	0.0847	0.1114	0.1962
	Asklepios	−17.73 (−21.97 to −13.49)	−46.05 (−52.76 to −39.33)	0.0257	0.0450	0.0708
	Combined	−17.22 (−19.40 to −15.04)	−39.44 (−42.08 to −36.80)	0.1058	0.0581	0.3967
Father’s age at birth (FAB)	UK Twins	13.39 (9.63 to 17.14)	−21.38 (−23.25 to −19.51)	0.0067	0.1560	0.1627
	NHLBI-FHS	14.56 (11.04 to 18.08)	−21.69 (−23.63 to −19.75)	0.0184	0.1774	0.1958
	Asklepios	17.17 (12.90 to 21.43)	−28.30 (−33.03 to −23.57)	0.0238	0.0450	0.0689
	Combined	14.85 (12.63 to 17.08)	−22.18 (−23.53 to −20.84)	0.0116	0.1629	0.3911

Multiple linear regression models using paternal birth year (PBY) in addition to the age, top panel, produce age-associated telomere shortening rates more consistent with longitudinal measurements of telomere shortening than models that utilize father’s age at birth (FAB), bottom panel, in all three datasets individually and combined. Further, models using PBY instead of age produce higher *R*^2^ values and have a lower fractional contribution from age.

Although models including PBY instead of FAB predicted telomere length better, the model including FAB and age substantially increased the predictive power over a model including age alone in all three datasets without yielding a large decrease in fractional *R*^2^ of age to the model. This suggests that both PBY and FAB impact telomere length independent of their collinearity and that including age, PBY, and FAB in subsequent models should improve their predictive power.

### Mediation analysis reveals age-associated telomere shortening rates consistent with longitudinal studies and the presence of a PBY effect distinct from FAB

**Fig 2:**
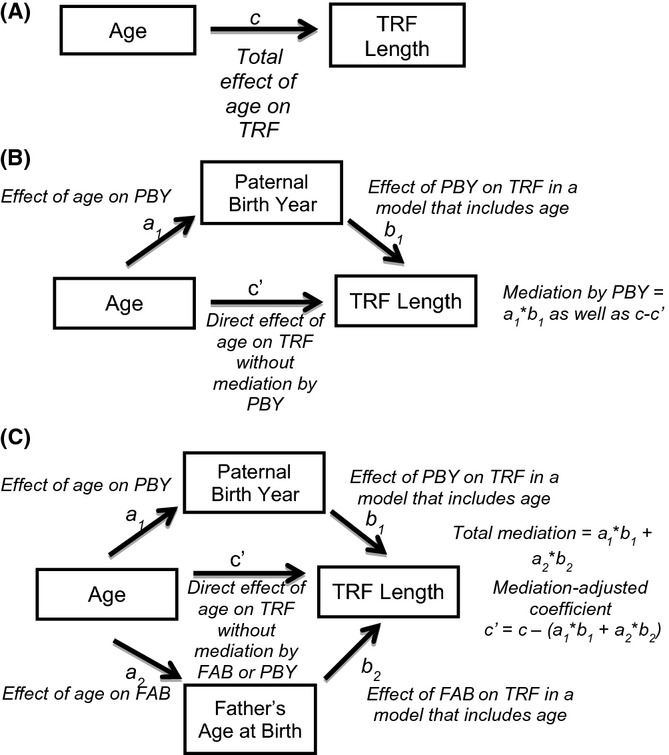
Method by which mediation analysis accounts for variable collinearity. Mediation analysis seeks to determine how much of an independent variable’s effect on a dependent variable is influenced by an interaction through a third variable, the mediator.

**Fig 3:**
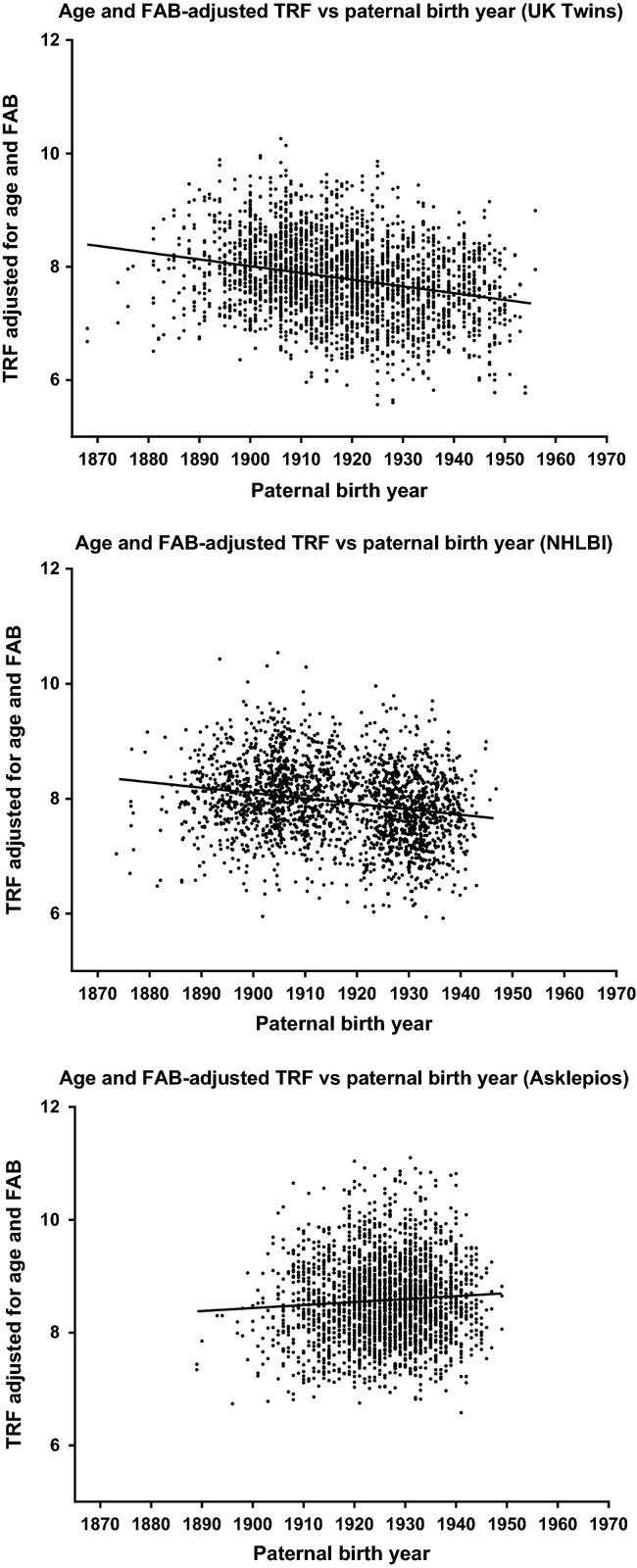
Paternal birth year effect in all three study populations. Age- and FAB-corrected telomere lengths demonstrate the paternal birth year effect in all three study populations.

Because of the observed multicollinearity of the three variables in question in all three datasets, we performed mediation analysis in order to understand how to correct for the indirect action of other variables (see Materials and methods and Fig.2 for a detailed explanation of mediation). Mediation-adjusted models for all three datasets and the combined data show age-associated shortening rates consistent with longitudinal studies (40.19 bp/year in the combined data) and larger FAB effects (38.62 bp/year) than previously reported (Table2). PBY effects were less pronounced and more heterogeneous compared to linear regression models. Both the UK Twins and the NHLBI-FHS dataset showed negative correlations between PBY and telomere length, while the Asklepios dataset indicated a positive correlation between PBY and telomere length (Fig.3). Sobel tests were performed on each mediation interaction to test for statistical significance (Table S4), and all interactions’ z-scores indicated *P*-values < 0.05.

**Table 2:** Mediation-adjusted models

Independent variable	Mediation (bp/year)			Total mediation	Adjusted coefficient
	Age	PBY	FAB		
UK Twins					
Age	N/A	20.26	0.70	20.95	−41.64
PBY	31.58	N/A	−7.10	24.47	−12.38
FAB	−4.50	−23.19	N/A	−27.69	36.58
NHLBI-FHS					
Age	N/A	15.12	0.39	15.51	−36.81
PBY	27.47	N/A	−8.72	18.75	−5.94
FAB	−1.90	−23.41	N/A	−25.31	37.97
Asklepios					
Age	N/A	20.12	2.37	22.49	−48.42
PBY	20.82	N/A	−24.02	−3.20	6.29
FAB	−14.08	−41.06	N/A	−55.14	58.23
Combined					
Age	N/A	18.00	0.74	18.75	−40.19
PBY	28.10	N/A	−10.04	18.06	−7.18
FAB	−3.05	−23.77	N/A	−26.82	38.62

The effect of each independent variable (age, PBY, and FAB) is adjusted for the effects of each other independent variable on telomere length and their collinearity within each dataset. The adjusted coefficients (far right) illustrate age-associated telomere shortening rates consistent with longitudinal observations, heterogeneity in PBY effects and reveal larger FAB effects than previously reported.

Segregating by offspring gender and repeating the mediation analysis revealed the same trends as considering the entire population as a whole (Tables S5 and S6) in all datasets with the exception of the UK Twins data, where a very small number of males (*N* = 199 males, 2511 females) complicate interpretation.

### Pseudo-longitudinal shortening rate in the UK Twins data is more consistent with mediation-adjusted age-associated shortening

The UK Twins dataset can be used to obtain another measurement of age-associated telomere shortening rate because not all twin pairs’ telomeres were measured at the same time. The difference in telomere length between twins in each twin pair is centered around zero for twins measured at the same time; however, as the time between visits increases, the difference between the first twin measured and the second twin measured increases due to age-associated telomere shortening. Because the twin measured second is older at the time of measurement than the twin measured first, this divergence in telomere length is due in part to age-associated telomere shortening. Although each measurement is a different individual, the mean intertwin variation is zero except for the effects of age and any variables that may contribute to delayed sampling of the second twin, such as socioeconomic status, illness, or travel. The difference in telomere length between the first twin and the second twin can therefore be viewed as a ‘pseudo-longitudinal’ telomere shortening assay. This pseudo-longitudinal shortening assay may give a more accurate picture of age-associated telomere shortening rate compared to a simple cross-sectional analysis because twins share both FAB and PBY.

**Fig 4:**
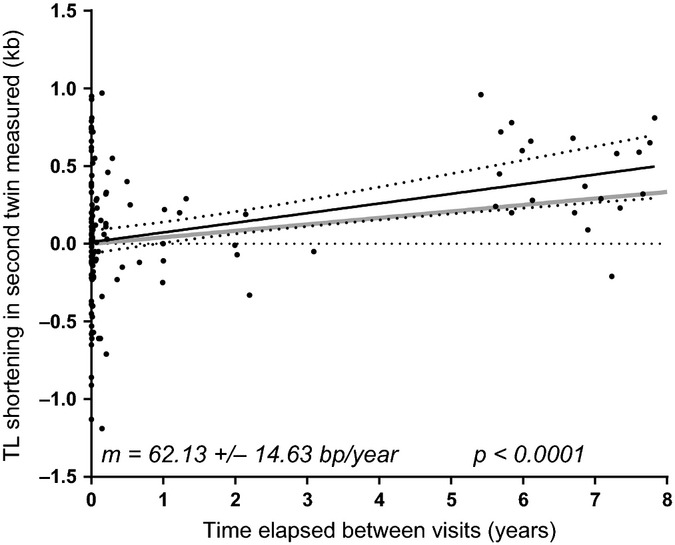
Pseudo-longitudinal measurements of telomere shortening in the UK Twins cohort. Because the difference in telomere length between the first and second twin measured is zero on average in twins measured at nearly the same time, the measurement of twins years apart may be considered a better measurement of telomere shortening rate. Pseudo-longitudinal telomere shortening in the UK Twins cohort is more consistent with the mediation-adjusted model (gray line) than simple cross-sectional analysis. The 95% confidence interval of the pseudo-longitudinal regression is demarcated by dotted lines.

Figure4 shows the difference in telomere length between the first twin measured and second twin measured for all twin pairs with different sample collection dates as a function of time elapsed between sample collection for the first and second twin measured. This indicates a telomere shortening rate of 62.13 bp/year. This shortening rate is substantially higher than the rate produced from the mediation-adjusted model, although that rate (gray line in Fig.4) falls within the 95% confidence interval for the pseudo-longitudinal regression.

### Correcting telomere length for age and paternal effects reveals a secular trend in initial telomere length

**Fig 5:**
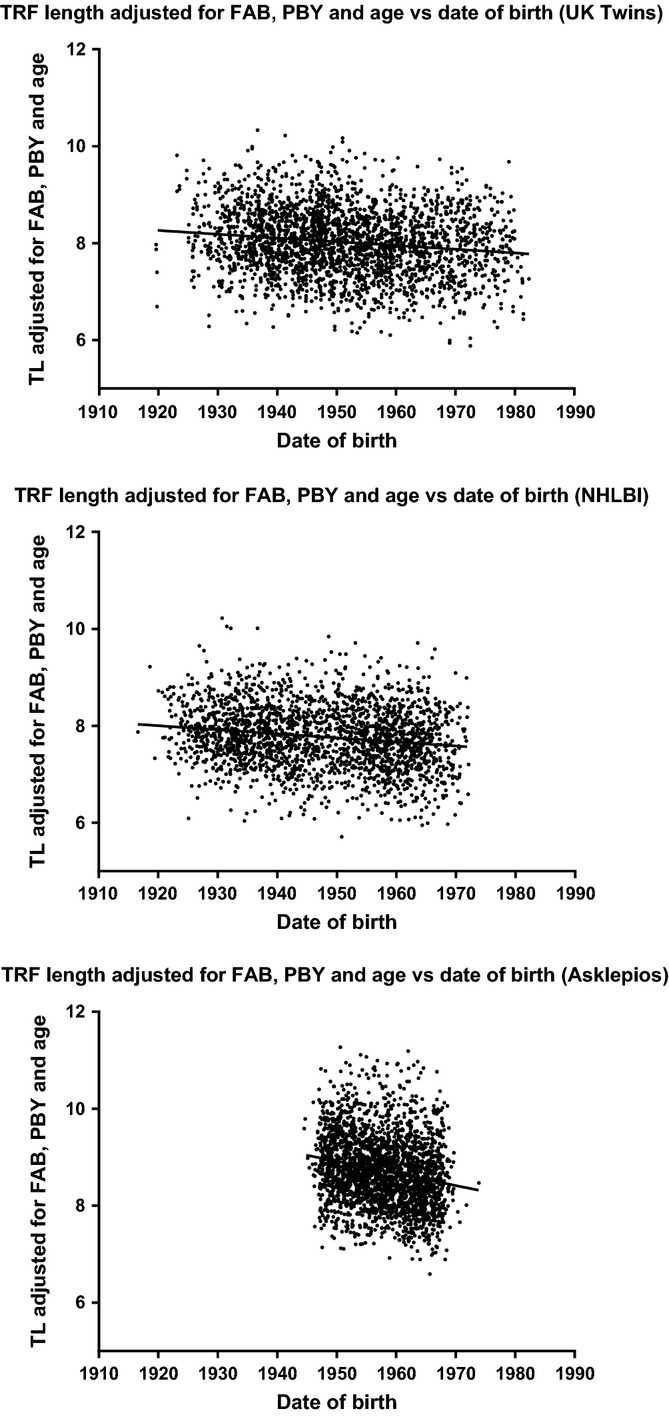
Changes in initial telomere length over time. Telomere length displays a negative correlation with date of birth after correcting for the mediation-adjusted effects of age, PBY, and FAB, showing that time-dependent influences on telomere length remain to be discovered.

Correcting telomere length for the mediation-adjusted effects of age, PBY, and FAB in each dataset, a calculation of initial telomere length after removing the effects of the paternal variables, reveals a time-dependent trend toward decreasing telomere length in relation to date of birth of 7.24, 14.97, and 23.80 bp/year in the UK Twins, NHLBI, and Asklepios data, respectively (Fig.5). As the mediation-adjusted effects account for the collinearity of the variables, the presence of a trend after correcting for these effects indicates the presence of at least one missing variable that impacts telomere length and has some relationship with time. As this trend was not accounted for by the PBY effect, this may represent the feed-forward effect predicted by the dual inheritance modality of telomere length or a relatively recent (occurring in the 1900s) environmental exposure affecting initial birth telomere length.

## Discussion

Because different species (Gomes *et al*., 2011) and human ethnic groups possess variable telomere lengths, average initial telomere length must be capable of change over evolutionary time. However, it was unknown whether a secular trend in initial telomere length was currently present in human populations and, if so, what may be driving such a change. We initially hypothesized that the FAB effect and the observed increase in sperm TL in older men may have been a marker for such a secular trend in initial telomere length, as both observations could have resulted from a decrease in initial telomere length over time. Our analysis revealed that although initial telomere length does appear to be changing in relation to PBY, the FAB effect becomes larger after accounting for the collinearity between FAB, PBY, and age. The mechanism for the progressive telomere lengthening in sperm cell progenitors that may be driving this FAB effect is a very interesting enigma, as an indefinite upward trend in telomere length has not been observed in telomerase-positive cells in tissue culture.

It is important to note that PBY is used here as a proxy variable for some unknown variable that has a relationship with time, not to indicate that PBY itself is driving such a change. The PBY effect appears to be highly variable between populations, and it is possible that this represents sensitivity to past differences in the environment, for instance in exposure to industrial pollution or malnutrition. Both datasets showing shortening in response to PBY were composed of heterogeneous, predominantly urban populations, while the population of the Asklepios study was composed of individuals living in two smaller communities in Belgium; it is possible that some urban environmental exposure may be driving this difference. Although technical differences in telomere length measurement may partially explain differences in mean telomere length between populations under study, the Asklepios cohort exhibited longer telomeres than both of the other datasets even after accounting for the study-specific effects of age, PBY, and FAB, suggesting that the difference in PBY effect may have existed for some time and this trend may explain why the Asklepios population has longer telomeres. It is also possible that some feature of sample selection in each study may be responsible for the difference in PBY effect, as the Asklepios population only included individuals that were free of overt cardiovascular disease while both of the other datasets did not exclude individuals based on their health, potentially biasing the trend observed in that cohort. The variability of the PBY effect may be one of the factors driving heterogeneity in telomere length between human groups, and the magnitude of this effect is large enough that it could yield relatively large changes in LTL over relatively short periods of time. For example, if the UK Twins population and Asklepios population started at the same telomere length and diverged only according to their PBY effect over time, they would be separated by 1 kb in mean telomere length in 53.5 years. It is likely that the PBY effect will vary both spatially and chronologically due to as-yet-undiscovered genetic, environmental, historical, and demographic drivers.

Particularly in the developed world, the prevalence of many environmental factors known to impact telomere length, such as those derived from traffic pollution and urban garbage (Hoxha *et al*., 2009; De Felice *et al*., 2012; Hou *et al*., 2012), changed dramatically in the past 200 years due to the industrial revolution, and this change may manifest as a change in telomere length at birth if those environmental changes alter telomere length in germ cells or during fetal development.

Accounting for the collinearity between the variables in the model and including PBY resolves the discrepancy in measured age-associated telomere shortening rate between cross-sectional and longitudinal studies, as the inclusion of both PBY and FAB in the model produces telomere shortening rates consistent with longitudinal studies in all three of these cross-sectional datasets. Many cross-sectional studies have reported heterogeneity in the effects of age and FAB, and it is possible that re-analysis of their data using this method accounting for PBY and the collinearity of these variables would clarify these relationships. The true heterogeneity in these effects remains poorly understood at this juncture because very few longitudinal studies have been conducted and most cross-sectional studies analyzed each effect without accounting for collinearity with other variables.

A secular trend, a negative relationship between initial telomere length and date of birth, exists in all three datasets even after removing the effects of age, FAB, and PBY, demonstrating that at least one additional variable is causing a reduction in initial telomere length over time. A maternal effect is one possibility, but it was not possible in the current study to evaluate maternal variables and paternal variables at the same time. If the paternal effects have existed for more than one generation (which is the case in at least one known population (Eisenberg *et al*., 2012)), maternal effects on telomere length are a logical consequence of the heritability of telomere length, as women would inherit altered telomere length from their fathers and pass this different initial telomere length to their offspring through their germ line. In addition to a feed-forward effect from paternally inherited shorter telomeres, maternal effects may be driven through environmental stresses to oocytes arrested in meiosis for decades that could damage telomeres and drive telomere shortening with advanced maternal age. There is some evidence that paternally transmitted telomeres are more important for determining telomere length in the offspring (Nordfjall *et al*., 2010), and inheriting mutant telomerase alleles paternally is more detrimental to telomere length compared to the same alleles transmitted maternally (Diaz de Leon *et al*., 2010), although the parent-specific heritability of telomere length is still not fully determined (Eisenberg, 2014). A maternal effect has not been definitively ruled out in the general population and at least some amount of telomere length is inherited maternally, as wild-type offspring of female TERT mutant heterozygotes inherit short telomeres from their mother, albeit to a lesser degree (Diaz de Leon *et al*., 2010).

The epigenetic aspects of telomere length heritability may be largely driven by male gametes (De Meyer & Eisenberg, 2014) because egg progenitor cells undergo far fewer divisions per generation compared to sperm progenitor cells that divide throughout the lifetime of a male. However, oocytes remain in meiotic arrest for decades and could also be subjected to environmental stressors that could result in shortening of telomeres in the absence of cell division via breakage. Because half the genome is inherited from the father, the FAB effect should be roughly half of the lengthening in sperm telomeres; the FAB effect reported in this model is substantially larger than half of the reported lengthening in sperm (57 bp/year). However, the measurement of lengthening in sperm may have been subject to the same biases of other cross-sectional analyses, for example not accounting for the collinearity in the sperm donors between age and important variables known to impact telomere length, such as FAB, nor for the previously unreported PBY effect. It is possible that the actual lengthening in sperm progenitor cells over time is substantially smaller than reported previously because the PBY and FAB trends observed in this analysis that reduce observed age-associated shortening should exacerbate cross-sectionally the observed lengthening of sperm telomere length. Longitudinal analysis of telomere dynamics in sperm should resolve the discrepancy, as a longitudinal analysis would not be subject to underlying shifts in initial telomere length. It is also possible that sperm telomeres are more important than oocyte telomeres in determining offspring telomere length, and the FAB effect is more than half of the lengthening observed in sperm as a result of this difference.

The causes of the PBY effect and secular trend in initial telomere length must be relatively new, as these trends would have pushed these populations into potentially pathological telomere length ranges, those observed in families with telomeropathies, 2–3 kb shorter (Alter *et al*., 2012), in a few centuries. Given that many of the environmental insults known to impact telomeres are the results of industrial pollution, the PBY effect and the secular trend in initial telomere length may be the cumulative result of many different evolutionarily novel insults to telomere maintenance. The presence of secular trends in many other parameters, such as obesity, height, pubertal onset, and smoking behavior may also be related to the secular trend in initial telomere length. However, many of these trends display substantial nonlinearity that is not observed in the secular trend in initial telomere length, suggesting the other secular trends may not be driving the change in telomere length. At the present time, it is unclear how long the observed trend in shorter initial telomere length has been occurring, although measurement of telomere length in archeological samples may be able to provide insight.

While the biological significance of the secular trend in reduced initial telomere length is not yet understood, there may be implications for public health. Correlations exist between short telomere length and a wide variety of pathologies; for example, robust inverse correlations exist between telomere length and asthma incidence and severity (Albrecht *et al*., 2014), and the incidence of asthma in the developed world has been rising precipitously in the recent past (Eder *et al*., 2006). Although it is not known whether the relationship between asthma and telomere length is causative or correlative, if it were partially causative, a decrease in initial telomere length in the population could increase asthma risk and may represent one cause for the increase in asthma incidence. Lungs seem to be one of the organs most sensitive to short telomere length and impaired telomere maintenance, with diseases such as idiopathic pulmonary fibrosis (IPF) and other lung pathology in the earliest generations of families with mutations in TERT (Diaz de Leon *et al*., 2011). Furthermore, short telomeres are protective for certain cancers and risk factors for other types of cancers (Wentzensen *et al*., 2011; Anic *et al*., 2013; Walcott *et al*., 2013), and a secular trend in initial telomere length may impact the frequencies of these cancers.

In conclusion, we have identified a change in initial telomere length linked to paternal birth year. In addition, we have shown how inclusion of PBY in the analysis of cross-sectional telomere datasets can reconcile the differences between cross-sectional and longitudinal measurements of telomere shortening. This reconciliation has refined our understanding of the age-associated rate of telomere shortening, and we presented evidence that longitudinal measurements are more likely to be correct. We have also shown that the effect of the father’s age at birth of their offspring on their offspring’s telomere length is larger than previously reported after accounting for FAB’s collinearity with other variables. Lastly, we have identified a downward secular trend in initial telomere length after accounting for the known age and paternal effects on telomere length that may have public health implications, which indicates that other influences on telomere length remain to be discovered.

## Experimental procedures

### Mediation analysis

The multilevel R package (Bliese, 2006) was used to perform the mediation analysis, and results were confirmed via the method of Preacher and Hayes (Preacher & Hayes, 2008). Mediation analysis is designed to disentangle the effects of collinear variables and allows quantitation of the amount of a given independent variable’s total effect on a dependent variable that is exerted via an indirect effect through at least one other variable, the mediator. Using the product-of-coefficients approach (Preacher & Hayes, 2008), the total effect of each independent variable (age, PBY, and FAB) on the dependent variable (TRF length) is determined by linear regression analysis independently (Fig.2A). The effect of each independent variable on each other independent variable is then determined, as are the effects of each independent variable on telomere (TRF) length in regression models that include one other independent variable (regression matrices are given in Table S2). The indirect effect of the independent variable through the mediator is then computed as the product of the independent variable’s effect on the mediator (a_1_) and the mediator’s effect on the dependent variable accounting for the independent variable (b_1_), which will also correspond to the difference between the independent variable’s effect on the dependent variable (c) and the independent variable’s effect on the dependent variable in a model that includes the mediator (c’, Fig.2B). This procedure can be carried out for any number of mediators to obtain an estimate of the direct effect of an independent variable accounting for its collinearity with mediator variables (Fig.2C).

### Statistics

Datasets were obtained from the groups that initially collected the samples and measured telomere length (Higgins *et al*., 1996; Andrew *et al*., 2001; De Meyer *et al*., 2007; Rietzschel *et al*., 2007; Kimura *et al*., 2008). All three datasets used the TRF assay Southern blot-based telomere measurement technique, facilitating interpopulation comparisons. Linear regressions and Pearson’s correlations were computed using the minitab 17 software package.

Age- and paternal effects-corrected telomere lengths used for Figures3 and 5 were computed by adjusting all telomere lengths using the mediation-adjusted coefficients from this work to age and father’s age at birth = 0 and paternal birth year = 1900.

## Supporting Information

**Figure S1.** Multicollinearity between FAB, PBY and Age.

**Table S1.** Comparison of cross-sectional and longitudinal measurements of telomere shortening. **Table S2.** Multicollinearity between FAB, PBY, MAB, MBY and Age in the combined datasets. **Table S3.** Regression matrices for mediation analysis. **Table S4.** Sobel tests for mediation. **Table S5.** Gender-segregated mediation analysis. **Table S6.** Sobel tests for gender-segregated mediation analysis.

**Video S1.** Illustration of how changing initial telomere length would bias cross-sectional telomere shortening rates. Cross-sectional analyses rely on the assumption that initial telomere length is not changing. If initial length is changing, observed shortening rates are biased in the direction opposite the change in initial telomere length, as the change in initial length is incorrectly ascribed to aging. The video shows birth and aging of individuals (blue circles) over time. Individuals are born at age zero and move to the right as they age; other individuals are born at later times and at differing initial telomere lengths, but all individuals shorten at the same rate over time. In the first model, initial telomere length does not change and the cross-sectional analysis, conducted when the solid line is drawn, yields a correct measurement of telomere shortening rate. Initial telomere length is increasing in the second model, which results in overestimation of the telomere shortening rate (solid line) at analysis compared with the actual shortening rate (dashed line). The third model shows how reduction in initial telomere length over time would yield an underestimate of telomere shortening rate at cross-sectional analysis.
