# Supplementary material for: Decreasing initial telomere length in humans intergenerationally understates age-associated telomere shortening
Source: Aging Cell. 2015 May 7;14(4):669–77. doi: 10.1111/acel.12347 (PMC4531080; doi:10.1111/acel.12347)
Supplement: Supplementary file 1 [file acel0014-0669-sd1.pdf]

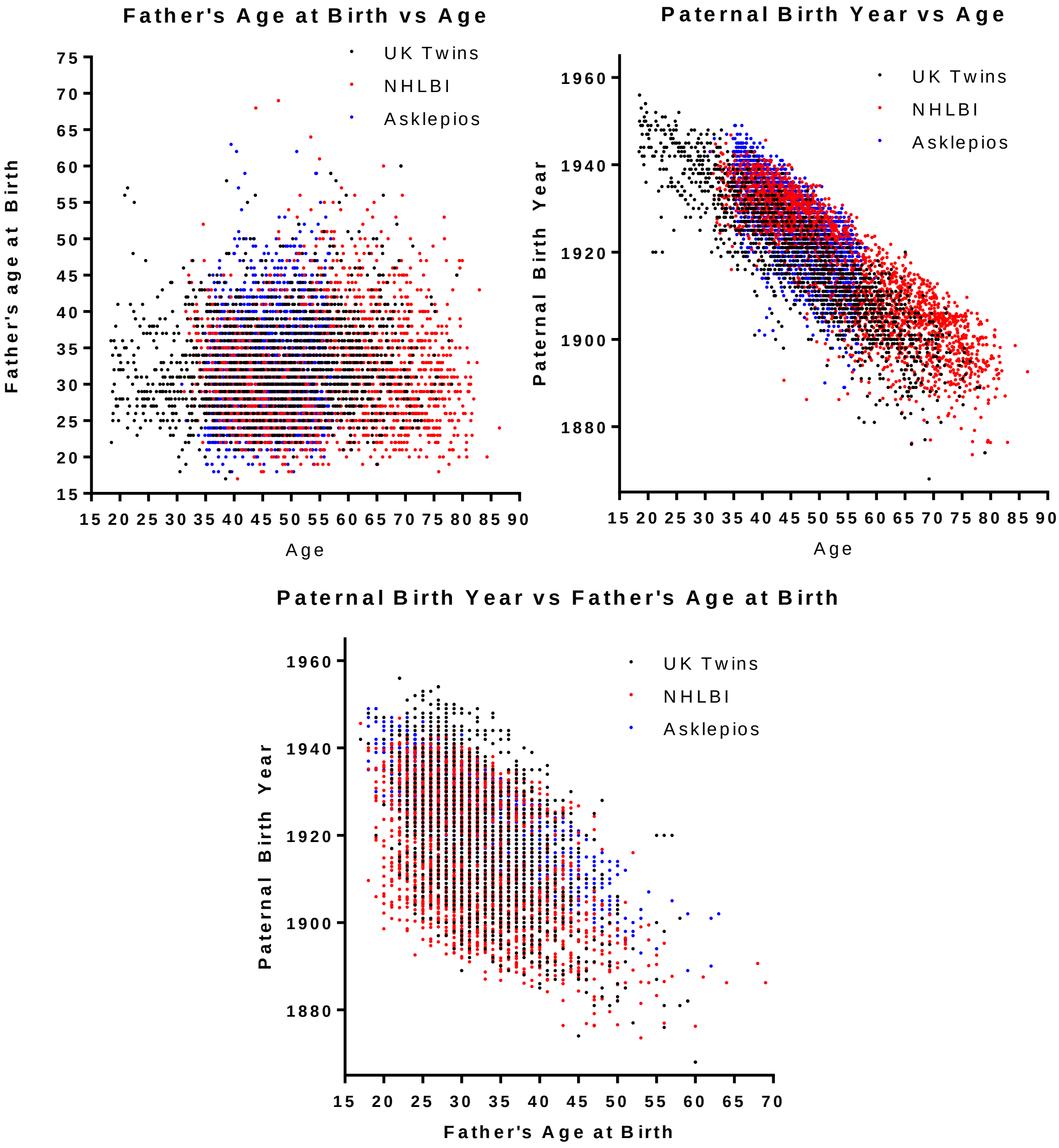
Father's Age at Birth vs Age
UK Twins
NHLBI
Asklepios
Father's age at Birth
75
70
65
60
55
50
45
40
35
30
25
20
15
15 20 25 30 35 40 45 50 55 60 65 70 75 80 85 90
Age
Paternal Birth Year vs Age
UK Twins
NHLBI
Asklepios
Paternal Birth Year
1960
1940
1920
1900
1880
15 20 25 30 35 40 45 50 55 60 65 70 75 80 85 90
Age
Paternal Birth Year vs Father's Age at Birth
UK Twins
NHLBI
Asklepios
Paternal Birth Year
1960
1940
1920
1900
1880
15 20 25 30 35 40 45 50 55 60 65 70
Father's Age at Birth
