# Supplementary material for: Decreasing initial telomere length in humans intergenerationally understates age-associated telomere shortening
Source: Aging Cell. 2015 May 7;14(4):669–77. doi: 10.1111/acel.12347 (PMC4531080; doi:10.1111/acel.12347)
Supplement: Supplementary file 2 [file acel0014-0669-sd2.docx]

References for Table S1:

1. Aviv A, Valdes A, Gardner JP, Swaminathan R, Kimura M, Spector TD. Menopause modifies the association of leukocyte telomere length with insulin resistance and inflammation. The Journal of clinical endocrinology and metabolism. 2006;91(2):635-40 DOI: 10.1210/jc.2005-1814.

2. Barwell J, Pangon L, Georgiou A, Docherty Z, Kesterton I, Ball J, et al. Is telomere length in peripheral blood lymphocytes correlated with cancer susceptibility or radiosensitivity? British journal of cancer. 2007;97(12):1696-700 DOI: 10.1038/sj.bjc.6604085.

3. Bataille V, Kato BS, Falchi M, Gardner J, Kimura M, Lens M, et al. Nevus size and number are associated with telomere length and represent potential markers of a decreased senescence in vivo. Cancer epidemiology, biomarkers & prevention : a publication of the American Association for Cancer Research, cosponsored by the American Society of Preventive Oncology. 2007;16(7):1499-502 DOI: 10.1158/1055-9965.EPI-07-0152.

4. Cherkas LF, Aviv A, Valdes AM, Hunkin JL, Gardner JP, Surdulescu GL, et al. The effects of social status on biological aging as measured by white-blood-cell telomere length. Aging cell. 2006;5(5):361-5 DOI: 10.1111/j.1474-9726.2006.00222.x.

5. Cherkas LF, Hunkin JL, Kato BS, Richards JB, Gardner JP, Surdulescu GL, et al. The association between physical activity in leisure time and leukocyte telomere length. Archives of internal medicine. 2008;168(2):154-8 DOI: 10.1001/archinternmed.2007.39.

6. Fitzpatrick AL, Kronmal RA, Kimura M, Gardner JP, Psaty BM, Jenny NS, et al. Leukocyte telomere length and mortality in the Cardiovascular Health Study. The journals of gerontology Series A, Biological sciences and medical sciences. 2011;66(4):421-9 DOI: 10.1093/gerona/glq224.

7. Hunt SC, Chen W, Gardner JP, Kimura M, Srinivasan SR, Eckfeldt JH, et al. Leukocyte telomeres are longer in African Americans than in whites: the National Heart, Lung, and Blood Institute Family Heart Study and the Bogalusa Heart Study. Aging cell. 2008;7(4):451-8 DOI: 10.1111/j.1474-9726.2008.00397.x.

8. Richards JB, Valdes AM, Gardner JP, Kato BS, Siva A, Kimura M, et al. Homocysteine levels and leukocyte telomere length. Atherosclerosis. 2008;200(2):271-7 DOI: 10.1016/j.atherosclerosis.2007.12.035.

9. Steer SE, Williams FM, Kato B, Gardner JP, Norman PJ, Hall MA, et al. Reduced telomere length in rheumatoid arthritis is independent of disease activity and duration. Annals of the rheumatic diseases. 2007;66(4):476-80 DOI: 10.1136/ard.2006.059188.

10. Valdes AM, Andrew T, Gardner JP, Kimura M, Oelsner E, Cherkas LF, et al. Obesity, cigarette smoking, and telomere length in women. Lancet. 2005;366(9486):662-4 DOI: 10.1016/S0140-6736(05)66630-5.

11. Aviv A, Chen W, Gardner JP, Kimura M, Brimacombe M, Cao X, et al. Leukocyte telomere dynamics: longitudinal findings among young adults in the Bogalusa Heart Study. American journal of epidemiology. 2009;169(3):323-9 DOI: 10.1093/aje/kwn338.

12. Chen W, Kimura M, Kim S, Cao X, Srinivasan SR, Berenson GS, et al. Longitudinal versus cross-sectional evaluations of leukocyte telomere length dynamics: age-dependent telomere shortening is the rule. The journals of gerontology Series A, Biological sciences and medical sciences. 2011;66(3):312-9 DOI: 10.1093/gerona/glq223.

13. Gardner JP, Li S, Srinivasan SR, Chen W, Kimura M, Lu X, et al. Rise in insulin resistance is associated with escalated telomere attrition. Circulation. 2005;111(17):2171-7 DOI: 10.1161/01.CIR.0000163550.70487.0B.

14. Ehrlenbach S, Willeit P, Kiechl S, Willeit J, Reindl M, Schanda K, et al. Influences on the reduction of relative telomere length over 10 years in the population-based Bruneck Study: introduction of a well-controlled high-throughput assay. International journal of epidemiology. 2009;38(6):1725-34 DOI: 10.1093/ije/dyp273.

15. Farzaneh-Far R, Lin J, Epel E, Lapham K, Blackburn E, Whooley MA. Telomere length trajectory and its determinants in persons with coronary artery disease: longitudinal findings from the heart and soul study. PloS one. 2010;5(1):e8612 DOI: 10.1371/journal.pone.0008612.

16. Houben JM, Giltay EJ, Rius-Ottenheim N, Hageman GJ, Kromhout D. Telomere length and mortality in elderly men: the Zutphen Elderly Study. The journals of gerontology Series A, Biological sciences and medical sciences. 2011;66(1):38-44 DOI: 10.1093/gerona/glq164.

17. Benetos A, Kark JD, Susser E, Kimura M, Sinnreich R, Chen W, et al. Tracking and fixed ranking of leukocyte telomere length across the adult life course. Aging cell. 2013;12(4):615-21 DOI: 10.1111/acel.12086.

| **Cross-sectional study** | **N** | **Shortening rate (bp/year)** |
| --- | --- | --- |
| Valdes, 2005 | 1122 | 27 |
| Aviv, 2006 | 1517 | 20.5 |
| Cherkas, 2006 | 1552 | 19.8 |
| Steer, 2007 | 1327 | 22 |
| Bataille, 2007 | 1897 | 27 |
| Barwell, 2007 | 1768 | 22 |
| Cherkas, 2008 | 2152 | 22 |
| Richards, 2008 | 1207 | 18.5 |
| Hunt, 2008 | 1395 | 20 |
| Fitzpatrick, 2011 | 1136 | 26 |
| Weighted average |  | 22.82 |
| **Longitudinal study** | **N** | **Shortening rate (bp/year)** |
| Gardner, 2005 | 70 | 31.3 |
| Ehrlenbach, 2009 | 510 | 45.5 |
| Aviv, 2009 | 685 | 40.7 |
| Farzaneh-Far, 2010 | 608 | 42 |
| Chen, 2011 | 271 | 31.6 |
| Houben, 2011 | 75 | 40.2 |
| Weighted average |  | 41.19 |

**Table S1**

**Cross-sectional vs. Longitudinal estimates of telomere shortening demonstrate underestimation of telomere shortening rate in cross-sectional measurements**

Telomere shortening rate as measured cross-sectionally is consistently ~20 bp/year slower than directly observed longitudinally. References for table S1 are given in supporting document 1.

| **Pearson Correlation** | | | | |
| --- | --- | --- | --- | --- |
| Variable | Age | PBY | FAB | MAB |
| PBY | -0.848* |  |  |  |
| FAB | 0.061* | -0.546* |  |  |
| MAB | 0.021 (NS) | -0.415* | 0.796* |  |
| MBY | -0.869* | 0.957* | -0.416* | -0.469* |

*: p <0.01

NS: Not Significant
PBY: Paternal Birth Year

FAB: Father’s Age at offspring’s Birth

MAB: Mother’s Age at offspring’s Birth

MBY: Maternal Birth Year

**Table S2.**

**Multicollinearity between FAB, PBY, MAB, MBY and Age in the combined datasets.**

| **Regression** | |  |  |  |
| --- | --- | --- | --- | --- |
| **Study** | **Dependent  Variable** | **Independent variable** | | |
|  |  | **Age** | **PBY** | **FAB** |
| UK Twins | Age (years/year) | * | -0.77121 | 0.2105 |
|  | PBY (years/year) | -1.0401 | * | -1.2077 |
|  | FAB (years/year) | 0.05205 | -0.22146 | * |
|  | TRF (kb/year) | -0.020683 | 0.012097 | 0.00889 |
| NHLBI | Age (years/year) | * | -0.7543 | 0.0875 |
|  | PBY (years/year) | -1.031 | * | -1.0871 |
|  | FAB (years/year) | 0.0266 | -0.24171 | * |
|  | TRF (kb/year) | -0.0213 | 0.01281 | 0.01266 |
| Asklepios | Age (years/year) | * | -0.45211 | 0.1124 |
|  | PBY (years/year) | -1.1344 | * | -1.1127 |
|  | FAB (years/year) | 0.1379 | -0.54418 | * |
|  | TRF (kb/year) | -0.02593 | 0.00309 | 0.00309 |

| **Standard Error** | |  |  |  |
| --- | --- | --- | --- | --- |
| **Study** | **Dependent  Variable** | **Independent variable** | | |
|  |  | **Age** | **PBY** | **FAB** |
| UK Twins | Age (years/year) | * | 0.00736 | 0.0384 |
|  | PBY (years/year) | 0.00993 | * | 0.0384 |
|  | FAB (years/year) | 0.00951 | 0.00704 | * |
|  | TRF (kb/year) | 0.000955 | 0.00086 | 0.00207 |
| NHLBI | Age (years/year) | * | 0.00865 | 0.0389 |
|  | PBY (years/year) | 0.0118 | * | 0.039 |
|  | FAB (years/year) | 0.0118 | 0.00868 | * |
|  | TRF (kb/year) | 0.001 | 0.000903 | 0.00198 |
| Asklepios | Age (years/year) | * | 0.00894 | 0.0182 |
|  | PBY (years/year) | 0.0224 | * | 0.0182 |
|  | FAB (years/year) | 0.0223 | 0.00891 | * |
|  | TRF (kb/year) | 0.00242 | 0.00156 | 0.00156 |

| **TRF Regression Coefficient** | **UK Twins (kb/year)** | **NHLBI (kb/year)** | **Asklepios (kb/year)** |  | **TRF Regression Standard Error** | **UK Twins (kb/year)** | **NHLBI (kb/year)** | **Asklepios (kb/year)** |
| --- | --- | --- | --- | --- | --- | --- | --- | --- |
|  |  |  |  |  |  |  |  |  |
| Age in Age+PBY | -0.04094 | -0.03642 | -0.04605 |  | Age in Age+PBY | 0.0021 | 0.0021 | 0.00342 |
| PBY in Age+PBY | -0.01948 | -0.01466 | -0.01773 |  | PBY in Age+PBY | 0.00181 | 0.00179 | 0.00216 |
| Age in Age+FAB | -0.02138 | -0.02169 | -0.0283 |  | Age in Age+FAB | 0.000952 | 0.00099 | 0.00241 |
| FAB in Age+FAB | 0.01339 | 0.01456 | 0.01717 |  | FAB in Age+FAB | 0.00191 | 0.0018 | 0.00218 |
| FAB in FAB+PBY | 0.03208 | 0.03607 | 0.04415 |  | FAB in FAB+PBY | 0.00226 | 0.00209 | 0.00345 |
| PBY in FAB+PBY | 0.0192 | 0.021528 | 0.02711 |  | PBY in FAB+PBY | 0.00097 | 0.00099 | 0.00241 |

**Table S3.**

**Regression matrices for mediation analysis.**

| UK Twins |  |  |  |
| --- | --- | --- | --- |
| **Independent  Variable** | **Mediation (z-score)** | | |
|  | **Age** | **PBY** | **FAB** |
| Age | * | 10.71 | 4.31 |
| PBY | 19.17 | * | -12.94 |
| FAB | -5.33 | -16.75 | * |
|  |  |  |  |
| NHLBI-FHS |  |  |  |
| **Independent  Variable** | **Mediation (z-score)** | | |
|  | **Age** | **PBY** | **FAB** |
| Age | * | 8.15 | 2.17 |
| PBY | 8.15 | * | -14.67 |
| FAB | -2.24 | -17.19 | * |
|  |  |  |  |
| Asklepios |  |  |  |
| **Independent  Variable** | **Mediation (z-score)** | | |
|  | **Age** | **PBY** | **FAB** |
| Age | * | 8.10 | 4.86 |
| PBY | 13.01 | * | -12.53 |
| FAB | -5.47 | -11.06 | * |
|  |  |  |  |
| Combined |  |  |  |
| **Independent  Variable** | **Mediation (z-score)** | | |
|  | **Age** | **PBY** | **FAB** |
| Age | * | 15.43 | 2.11 |
| PBY | 31.57 | * | -7.41 |
| FAB | -6.97 | -26.95 | * |

**Table S4.**

**Sobel tests for mediation**

| UK Twins (F) |  |  |  |  |  |
| --- | --- | --- | --- | --- | --- |
| **Independent  Variable** | **Mediation (bp/year)** | | | **Total  Mediation** | **Adjusted  Coefficient** |
|  | **Age** | **PBY** | **FAB** |  |  |
| Age | * | 18.91 | 0.72 | 19.63 | -40.62 |
| PBY | 30.80 | * | -7.47 | 23.33 | -10.70 |
| FAB | -4.51 | -24.17 | * | -28.68 | 37.97 |
| UK Twins (M) |  |  |  |  |  |
| **Independent  Variable** | **Mediation (bp/year)** | | | **Total  Mediation** | **Adjusted  Coefficient** |
|  | **Age** | **PBY** | **FAB** |  |  |
| Age | * | 2.19 | -0.33 | 1.86 | -15.37 |
| PBY | 12.30 | * | -0.90 | 11.40 | -1.20 |
| FAB | -3.66 | -14.14 | * | -17.80 | 8.26 |
| NHLBI (F) |  |  |  |  |  |
| **Independent  Variable** | **Mediation (bp/year)** | | | **Total  Mediation** | **Adjusted  Coefficient** |
|  | **Age** | **PBY** | **FAB** |  |  |
| Age | * | 15.14 | 0.50 | 15.64 | -37.26 |
| PBY | 27.89 | * | -8.63 | 19.26 | -5.93 |
| FAB | -2.64 | -24.55 | * | -27.19 | 39.09 |
| NHLBI (M) |  |  |  |  |  |
| **Independent  Variable** | **Mediation (bp/year)** | | | **Total  Mediation** | **Adjusted  Coefficient** |
|  | **Age** | **PBY** | **FAB** |  |  |
| Age | * | 14.88 | 0.25 | 15.13 | -36.54 |
| PBY | 27.19 | * | -8.91 | 18.28 | -5.68 |
| FAB | -1.15 | -22.67 | * | -23.82 | 37.12 |
| Asklepios (F) |  |  |  |  |  |
| **Independent  Variable** | **Mediation (bp/year)** | | | **Total  Mediation** | **Adjusted  Coefficient** |
|  | **Age** | **PBY** | **FAB** |  |  |
| Age | * | 22.12 | 2.89 | 25.01 | -45.72 |
| PBY | 19.89 | * | -22.31 | -2.42 | 3.07 |
| FAB | -3.06 | -25.95 | * | -29.01 | 45.07 |
| Asklepios (M) |  |  |  |  |  |
| **Independent  Variable** | **Mediation (bp/year)** | | | **Total  Mediation** | **Adjusted  Coefficient** |
|  | **Age** | **PBY** | **FAB** |  |  |
| Age | * | 18.37 | 1.892 | 37.29 | -51.23 |
| PBY | 21.64 | * | -25.92 | -4.28 | 9.46 |
| FAB | -3.09 | -34.04 | * | -37.13 | 49.49 |

**Table S5**

**Gender-segregated mediation analysis**

| UK Twins (F) |  |  |  |
| --- | --- | --- | --- |
| **Independent  Variable** | **Mediation (z-score)** | | |
|  | **Age** | **PBY** | **FAB** |
| Age | * | 9.81 | 4.23 |
| PBY | 18.33 | * | -13.26 |
| FAB | -5.10 | -17.02 | * |
| UK Twins (M) |  |  |  |
| **Independent  Variable** | **Mediation (z-score)** | | |
|  | **Age** | **PBY** | **FAB** |
| Age | * | 0.30 | -0.72 |
| PBY | 1.97 | * | -0.53 |
| FAB | -1.61 | -3.02 | * |
| NHLBI (F) |  |  |  |
| **Independent  Variable** | **Mediation (z-score)** | | |
|  | **Age** | **PBY** | **FAB** |
| Age | * | 5.70 | 2.06 |
| PBY | 12.14 | * | -10.57 |
| FAB | -2.19 | -12.70 | * |
| NHLBI (M) |  |  |  |
| **Independent  Variable** | **Mediation (z-score)** | | |
|  | **Age** | **PBY** | **FAB** |
| Age | * | 5.88 | 0.94 |
| PBY | 12.31 | * | -10.40 |
| FAB | -0.95 | -11.91 | * |
| Asklepios (F) |  |  |  |
| **Independent  Variable** | **Mediation (z-score)** | | |
|  | **Age** | **PBY** | **FAB** |
| Age | * | 6.11 | 3.88 |
| PBY | 8.54 | * | -8.42 |
| FAB | -4.07 | -6.76 | * |
| Asklepios (M) |  |  |  |
| **Independent  Variable** | **Mediation (z-score)** | | |
|  | **Age** | **PBY** | **FAB** |
| Age | * | 5.48 | 3.01 |
| PBY | 9.97 | * | -9.47 |
| FAB | -3.46 | -8.91 | * |

**Table S6.**

**Sobel tests for gender-segregated mediation analysis**
